# Bloqueio de Receptores AT_1_ Melhora o Desempenho Funcional Miocárdico na Obesidade

**DOI:** 10.36660/abc.20190131

**Published:** 2020-07-28

**Authors:** Silvio Assis de Oliveira-Junior, Nayara de Araújo Muzili, Marianna Rabelo de Carvalho, Gabriel Elias Ota, Camila Souza de Morais, Larissa Fregapani da Costa Vieira, Mateus Oliveira Ortiz, Dijon H. S. Campos, Marcelo Diacardia Mariano Cezar, Marina Politi Okoshi, Katashi Okoshi, Antonio C. Cicogna, Paula Felippe Martinez

**Affiliations:** 1 Universidade Federal de Mato Grosso do Sul Campo GrandeMS Brasil Universidade Federal de Mato Grosso do Sul, Campo Grande, MS – Brasil; 2 Faculdade de Medicina de Botucatu UNESP BotucatuSP Brasil Faculdade de Medicina de Botucatu – UNESP, Botucatu, SP - Brasil; 3 Faculdade de Ciências Sociais e Agrárias de Itapeva ItapevaSP Brasil Faculdade de Ciências Sociais e Agrárias de Itapeva (FAIT), Itapeva, SP - Brasil; 4 Universidade Estadual de São Paulo Departamento de Medicina Interna BotucatuSP Brasil Universidade Estadual de São Paulo (UNESP), Departamento de Medicina Interna, Botucatu, SP - Brasil; 5 Universidade Estadual Paulista Júlio de Mesquita Filho Faculdade de Medicina BotucatuSP Brasil Universidade Estadual Paulista Júlio de Mesquita Filho - Faculdade de Medicina - Campus de Botucatu, Botucatu, SP - Brasil

**Keywords:** Doenças Cardiovasculares, Obesidade, Losartan/uso terapêutico, Bloqueadores do Receptor Tipo 1 de Angiotensina II/uso terapêutico, Ratos, Dieta Hiperlipídica/métodos

## Abstract

**Fundamento:**

A obesidade tem sido associada com ativação crônica do sistema renina-angiotensina-aldosterona e importantes alterações no desempenho cardíaco.

**Objetivo:**

Avaliar a influência do bloqueio de receptores de angiotensina-II do tipo 1 (AT_1_) sobre a morfologia e desempenho cardíaco de ratos obesos por dieta

**Métodos:**

Ratos Wistar (n=48) foram submetidos a dieta controle (2,9 kcal/g) ou hiperlipídica (3,6 kcal/g) durante 20 semanas. Após a 16ª semana, foram distribuídos em quatro grupos: Controle (CO), Obeso (OB), Controle Losartan (CL) e Obeso Losartan (OL). CL e OL receberam losartan (30 mg/kg/dia) na água durante quatro semanas. Posteriormente, foram analisadas composição corporal, pressão arterial sistólica (PAS) e ecocardiograma. A função de músculos papilares foi avaliada em situação basal com concentração de cálcio ([Ca^2+^]_o_) de 2,50 mM e após manobras inotrópicas: potencial pós-pausa (PPP), elevação da [Ca^2+^]_o_ e durante estimulação beta-adrenérgica com isoproterenol. A análise dos resultados foi feita por meio de *Two-Way* ANOVA e teste de comparações apropriado. O nível de significância considerado foi de 5%.

**Resultados:**

Embora a alteração da PAS não tenha se mantido ao final do experimento, a obesidade se associou com hipertrofia cardíaca e maior velocidade de encurtamento da parede posterior do ventrículo esquerdo.No estudo de músculos papilares em condição basal, CL mostrou menor velocidade máxima de variação negativa da tensão desenvolvida (-dT/dt) do que CO. O PPP de 60s promoveu menor -dT/dt e pico de tensão desenvolvida (TD) em OB e CL, comparados ao CO, e maior variação relativa de TD e velocidade máxima de variação positiva (+dT/dt) no OL em relação a CL e OB. Sob 1,5, 2,0 e 2,5mM de [Ca^2+^]_o_, o grupo OL exibiu maior -dT/dt do que CL.

**Conclusão:**

Losartan melhora a função miocárdica de ratos com obesidade induzida por dieta. (Arq Bras Cardiol. 2020; 115(1):17-28)

## Introdução

A obesidade é uma doença crônica e multifatorial, resultante da interação entre diferentes fatores etiológicos.^1 , 2^ Configura uma disfunção nutricional e metabólica, que pode ser associada a dislipidemia, resistência à insulina e doenças cardiovasculares.^3^ Estudos clínicos demonstraram que a obesidade pode ocasionar alterações morfológicas e funcionais no coração.^4 , 5^ Pesquisas experimentais mostraram que essa condição se associa com hipertrofia miocárdica,^6 - 8^ fibrose intersticial,^8 , 9^ além de várias mudanças moleculares.^10 , 11^ Entre essas respostas, incluem-se alterações na expressão e funcionamento de peptídeos envolvidos com trânsito intracelular de cálcio, durante o processo de contração e relaxamento muscular.^7 , 12 - 14^

Contudo, há importantes divergências entre investigações com relação às repercussões da obesidade sobre desempenho miocárdico. Jacobsen et al.,^15^ constataram aumento na fase contrátil durante manobra inotrópica no músculo papilar em ratos obesos após três semanas de dieta; outros autores encontraram maior velocidade de encurtamento miocárdico em modelos de 20,^8^ 30,^11^ 33,^13^ e 35 semanas^16^ de duração. Outras investigações documentaram prejuízo na contração cardíaca, demonstrado por análises *in vitro* de músculos papilares de ratos obesos, após 15 semanas de dieta.^7 , 17 , 18^ Há também relatos de função cardíaca inalterada após 20,^9^ 30^19^ e 32^14^ semanas de dieta para indução de obesidade. Portanto, tem-se que o desempenho cardíaco necessita ser melhor estudado na obesidade induzida por dieta.

A condição de obesidade está relacionada com maior atividade do sistema renina-angiotensina-aldosterona (SRAA).^11 , 20 , 21^ Altos níveis de angiotensina-II (Ang-II) acoplando a receptores do tipo I (AT_1_) exercem um efeito vasoconstritor e trófico sobre o miocárdio, estimulando várias cascatas de sinalização intracelular e múltiplas respostas fisiológicas.^21 - 23^ A ativação do SRAA é o principal mecanismo responsável por distúrbios de pressão arterial e remodelação cardíaca na obesidade; essas desordens foram amenizadas após antagonismo de AT_1_.^6 , 11 , 21 , 24^ Entretanto, não foram encontrados estudos que tenham documentado a associação entre ativação do SRAA e remodelação ventricular na obesidade, considerando-se a análise *in vitro* do músculo papilar.

A preparação *in vitro* do músculo papilar permite mensurações da capacidade contrátil do miocárdio, em termos de encurtamento e geração de força, a despeito de alterações na carga, frequência cardíaca e geometria do coração, condições estas que modificam o desempenho mecânico in vivo.^7 , 13 , 17 , 19^ Com a utilização de manobras inotrópicas e lusinotrópicas, pode-se também estudar o desempenho miocárdico para identificar alterações na contração e no relaxamento, que não poderiam ser observadas em condições basais. As manobras mais comumente utilizadas são: potencial pós-pausa, elevação de Ca^2^ extracelular e estimulação beta-adrenérgica.^7^

Nessa perspectiva, o objetivo deste trabalho foi avaliar a influência do bloqueio de AT_1_ sobre a morfologia e desempenho cardíaco, utilizando-se de análise *in vitro* do músculo papilar, em ratos obesos por dieta hiperlipídica, com predomínio de ácidos graxos saturados. Como hipótese inicial, admite-se que a obesidade se associa com alterações no desempenho funcional miocárdico, sustentadas em diferentes condições de estimulação; essas respostas são amenizadas com o antagonismo de receptores AT_1_.

## Métodos

### Animais e delineamento experimental

Foram utilizados ratos *Wistar* machos (n=48), com 30 dias de idade, procedentes do Biotério Central da Universidade Estadual Paulista, campus de Botucatu/SP, Brasil. A definição do tamanho amostral foi baseada em estudo prévio,^19^ desenvolvido com modelo experimental similar e análise funcional do músculo papilar isolado.

Os animais foram distribuídos em dois grupos: controle (CO), tratado com dieta normolipídica (2,9 kcal/g), e obeso (OB), alimentado com dieta hiperlipídica, com predomínio de ácidos graxos saturados (3,6 kcal/g).^9^ Os seguintes ingredientes foram utilizados para ambas as preparações dietéticas: milho integral, proteína de soja, dextrina, óleos de soja e palma, vitaminas e minerais. Em termos de composição de ácidos graxos saturados/ insaturados, a dieta normolipídica apresentava 61,6/38,4%, respectivamente, enquanto a dieta hiperlipídica tinha 64,8/35,2%, respectivamente.^9 , 16^

Após 16 semanas, os animais foram alocados em quatro grupos: CO, OB, CL e OL. Durante mais quatro semanas, enquanto CO e OB continuaram recebendo as respectivas dietas, CL e OL receberam também losartan na água ingerida (30 mg/kg/dia).^11^ Os animais foram mantidos em gaiolas individuais, em temperatura ambiente de 22±2°C, umidade de 55±5% e ciclos de iluminação claro/escuro de 12 horas. O protocolo experimental foi analisado e aprovado pelo Comitê de Ética no Uso de Animais (CEUA/ UNESP; Protocolo 1000/2013)

### Estudo cardiovascular

O estudo cardiovascular envolveu mensuração da pressão arterial sistólica (PAS), avaliação da morfologia cardíaca, análise funcional por ecocardiograma e ensaio in vitro com músculo papilar. A análise da PAS e ecocardiograma foram feitos na 16ª e 20ª semana de experimento. A PAS foi obtida por meio de pletismografia,^26^ utilizando-se esfigmomanômetro ( *Narco Bio-Systems* ®, modelo 709-0610 - *International Biomedical* , Austin, TX, USA). Para o ecocardiograma, os animais foram anestesiados com uma mistura de cloridrato de cetamina (50 mg/kg) e cloridrato de xilidino (1 mg/kg), administrados por via intramuscular. Após a tricotomia na região anterior do tórax, cada animal foi posicionado em decúbito lateral esquerdo. Para análise da geometria cardíaca, foram obtidas imagens em modo monodimensional (modo-M) com o feixe de ultrassom orientado em modo bidimensional, mantendo-se o transdutor em posição paraesternal, eixo menor. A imagem do ventrículo esquerdo (VE) foi obtida posicionando-se o cursor do modo-M abaixo do plano da valva mitral no nível dos músculos papilares.^27^ As imagens da aorta e do átrio esquerdo (AE) foram obtidas com o cursor de modo-M posicionado ao nível do plano aórtico. As imagens foram registradas em impressora (modelo UP-890, Sony Co.). As estruturas cardíacas foram medidas manualmente com o auxílio de um paquímetro. No momento de diâmetro máximo da cavidade ventricular, foram mensurados o diâmetro diastólico do VE (DDVE), e espessuras diastólicas da parede posterior do VE (EDPP) e do septo interventricular (EDSIV). No momento de diâmetro mínimo da cavidade, foi avaliado o diâmetro sistólico do VE (DSVE). O AE foi medido no momento de seu diâmetro máximo. A massa do VE (MVE) foi calculada de acordo com a seguinte fórmula: MVE = [(DDVE+EDPP+EDSIV)^3^-(DDVE)^3^]x1,04. Considerou-se também a razão entre DDVE e comprimento da tíbia.

A função sistólica do VE foi avaliada pela velocidade de encurtamento da parede posterior (VEPP) e porcentagem de encurtamento endocárdico (%Enc.Endo): [(DDVE - DSVE)/DDVE]. A função diastólica foi analisada pelos seguintes índices: 1) razão entre os picos de velocidade de fluxo de enchimento inicial (onda E) e da contração atrial (onda A) do fluxo transmitral (E/A); 2) tempo de desaceleração da onda E (TDE); 3) tempo de relaxamento isovolumétrico (TRIV); 4) pico de velocidade de deslocamento diastólico inicial do anel mitral (E’) e pico de velocidade de deslocamento diastólico tardio do anel mitral (A’) obtidas pelo Doppler tissular; 5) razão entre as ondas E e E’ (E/E’). Todas as medidas foram feitas pelo mesmo pesquisador, conforme procedimentos da *American Society of Echocardiography* ,^28^ utilizando-se um ecocardiógrafo ( *General Electric Medical Systems, Vivid S6, Tirat Carmel* , Israel), equipado com transdutor eletrônico multifrequencial (5-11,5 MHz).

### Caracterização geral e análise da função miocárdica in vitro

A ingestão calórica foi avaliada diariamente.^6 - 8^ A eficiência alimentar foi obtida a partir da relação entre variação de massa corporal e energia total ingerida.^6 - 8^ A massa corporal foi mensurada semanalmente, enquanto o ganho de massa foi obtido a partir da diferença entre os valores de massa corporal inicial e final. O tecido adiposo das regiões retroperitoneal, epididimal e visceral foi utilizado para determinação do conteúdo de gordura corporal.^6 - 12^

A análise da função miocárdica foi realizada por ensaio *in vitro* com músculo papilar isolado do VE.^7 , 16 , 18 , 29^ Após 20 semanas, os animais foram submetidos a anestesia intraperitoneal com cloridrato de cetamina (80 mg/Kg) e xilazina (5 mg/Kg) e eutanásia. Após toracotomia mediana, o coração foi prontamente removido e dissecado. As massas de átrios (MA) e dos ventrículos direito (MVD) e esquerdo (MVE) foram utilizadas para análise macroscópica. Os músculos papilares dissecados do VE foram presos entre dois anéis de aço inoxidável e colocados verticalmente em uma câmara de vidro contendo solução de Krebs-Henseleit a 28°C, continuamente oxigenada com O_2_ (95%) e CO_2_ (5%). A composição da solução de Krebs foi a seguinte: 118,5 mM NaCl; 4,69 mM KCl; 2,50 mM CaCl_2_; 1,16 mM MgSO_4_; 1,18 mM KH_2_PO_4_; 5,50 mM glicose; e 24,88 mM NaCO_3_. A extremidade inferior do anel foi acoplada a um transdutor de força 120T-20B ( *Kyowa* , Tóquio, Japão) por um fio de aço (1/15,000) que atravessava uma fenda preenchida por mercúrio, existente no assoalho da câmara de vidro.^7 , 16 , 18 , 29^

Os músculos foram contraídos isotonicamente mediante carga leve por 60 minutos; a seguir, esses foram colocados em contração isométrica e estirados gradualmente até a tensão máxima desenvolvida (TD) atingir seu valor máximo. Após 5 minutos sob contrações isotônicas, os músculos foram colocados novamente em contração isométrica para determinação do pico da curva comprimento-tensão (Lmax). O comportamento dos músculos papilares foi avaliado em situação basal com concentração de cálcio ([Ca^2^]_o_) de 2,50 mM e após manobras inotrópicas: potencial pós-pausa (PPP), elevação da [Ca^2^]_o_ extracelular de 0,5 até 2,5 mM e durante estimulação beta-adrenérgica com 0,1 e 1,0 mM de isoproterenol. O potencial pós-pausa foi estudado em [Ca^2^]_o_ extracelular de 1,25 mM, em que o estímulo foi cessado por 30 e 60 segundos, antes de ser reiniciado.^7 , 30^

Depois do PPP, a resposta do músculo papilar foi avaliada após manobra extracelular da [Ca^2^]_o_.^31^ Os parâmetros contráteis isométricos foram registrados em 10 minutos após adição progressiva de cálcio (0,5 até 2,5mM) na solução extracelular. Além disso, a estimulação do sistema beta-adrenérgico foi também estudada para testar a integridade do complexo beta-adrenérgico, a sensibilidade da troponina C e a absorção de cálcio pelo retículo sarcoplasmático.^7 , 31^ A estimulação dos receptores adrenérgicos beta foi induzida utilizando-se concentrações cumulativas de isoproterenol (0,1 e 1,0 mM) na presença da [Ca^2^]_0_ de 1,0 mM.

### Estimadores mecânicos

Respostas mecânicas convencionais em Lmax foram obtidas em contração isométrica: tensão máxima desenvolvida normalizada pela área seccional transversa do músculo papilar (TD [g/mm^2^]) e velocidades máximas de variação positiva (+dT/dt [g/mm^2^/s]) e negativa (-dT/dt [g/mm^2^/s]) da TD, normalizada pela área seccional transversa do músculo papilar. Os estimadores usados para caracterizar o tamanho dos músculos papilares foram comprimento (mm), massa do músculo (mg) e área seccional transversa (AST [mm^2^]). Ao término de cada experimento, o comprimento do músculo em Lmax foi mensurado com auxílio de um catetômetro Gaertner ( *Gaertner Scientific Corporation* , Chicago, IL, USA), e a porção do músculo entre os anéis de aço foi cortada e pesada. A AST foi calculada dividindo-se a massa muscular pelo seu comprimento, assumindo uniformidade e uma gravidade específica de 1,0.

### Análise estatística

Para a análise dos dados, utilizou-se o programa Sigma Stat, versão 3.5. Inicialmente, os resultados foram submetidos a análise de normalidade por meio do teste de Kolmogorov-Smirnov. Como as variáveis tiveram distribuição paramétrica, os resultados foram apresentados em média e desvio-padrão. Para análise dos resultados nutricionais, composição corporal, morfologia cardíaca e desempenho funcional do músculo papilar, foi feita análise de variância de duas vias ( *Two-Way* ANOVA) e teste de comparações múltiplas de Tukey. As medidas de PAS e ecocardiograma foram analisadas por meio de *Two-Way* ANOVA no modelo de medidas repetidas (RM)e teste de comparações múltiplas de Bonferroni. O nível de significância considerado foi de 5%.

## Resultados

Os dados de comportamento nutricional, composição corporal e morfologia macroscópica do coração estão na Tabela 1 . Embora ingestão calórica não tenha sido alterada, OB e OL mostraram maior ingestão de gordura e eficiência alimentar do que CO e CL, respectivamente. A obesidade foi caracterizada por maiores medidas de massa e adiposidade corporal.

**Tabela 1:** – Média e desvio-padrão de variáveis de perfil nutricional, murinometria e morfologia cardíaca macroscópica, segundo o grupo

Variável		Grupo			

		CO	OB	CL	OL
**Perfil Nutricional**	**Massa Corporal (g)**	451 ± 58	507 ± 64 *	456 ± 49	517 ± 50 ^‡^
	**Ing. Calórica (Kcal)**	81,9 ± 8,2	80,7 ± 7,5	80,3 ± 9,2	78,7 ± 7,9
	**Ing. G. Insaturada (g)**	122 ± 12	235 ± 22 *	120 ± 14	230 ± 23 ^‡^
	**Ing. G. Saturada (g)**	196 ± 20	433 ± 40 *	193 ± 22	422 ± 43 ^‡^
	**Ef. Alimentar (%)**	26,82 ± 2,11	32,22 ± 4,38 *	28,43 ± 0,94	35,12 ± 4,78 ^‡^
	**Adiposidade (%)**	3,48 ± 0,73	5,19 ± 1,47 *	3,61 ± 1,27	5,50 ± 1,48 ^‡^
**Morfologia Macroscópica**	**Átrios (g)**	0,096 ± 0,015	0,113 ± 0,015 *	0,092 ± 0,009	0,100 ± 0,022 ^†^
	**Átrios/Tíbia (mg/mm)**	2,22 ± 0,33	2,59 ± 0,35 *	2,15 ± 0,21	2,29 ± 0,46 ^†^
	**MVD (g)**	0,231 ± 0,029	0,241 ± 0,030	0,230 ± 0,039	0,245 ± 0,040
	**MVD/Tíbia (mg/mm)**	5,36 ± 0,68	5,50 ± 0,70	5,34 ± 0,79	5,64 ± 0,86
	**MVE (g)**	0,844 ± 0,083	0,950 ± 0,099	0,800 ± 0,082 *	0,799 ± 0,087 ^†^
	**MVE/Tíbia (mg/mm)**	19,6 ± 1,7	21,7 ± 2,4 *	18,7 ± 1,5	18,4 ± 1,7 ^†^

Ing. Calórica: ingestão calórica; Ing. G. Insaturada: ingestão total de gordura insaturada; Ing. G. Saturada: ingestão total de gordura saturada; Ef. Alimentar: eficiência alimentar; MVD: massa de ventrículo direito; MVE: massa de ventrículo esquerdo; CO: grupo Controle; OB: grupo Obeso; CL: grupo controle sob losartan; OL: grupo obeso sob losartan. Efeitos de grupo: * p<0,05 comparado ao CO; † p<0,05 comparado ao OB; ‡ p<0,05 comparado ao CL; Two-Way ANOVA e teste de Tukey.

Quanto à morfologia cardíaca, comparado ao CO, o OB apresentou maiores valores de massa de átrios e das respectivas relações entre massa de átrios e VE com o comprimento tibial. O losartan repercutiu em menores medidas de átrios e VE, em valores absolutos e normalizados pelo comprimento da tíbia, no OL comparado ao OB ( Tabela 1 ).

Na Tabela 2 , são apresentados os resultados de PAS, bem como estrutura e desempenho do coração, avaliados por meio de ecocardiograma. Após 16 semanas, a obesidade esteve associada com maior PAS; o losartan culminou em redução da PAS no CL e OL, ao final do experimento. A razão entre diâmetro diastólico de ventrículo esquerdo (DDVE) e comprimento da tíbia não foi diferente entre grupos e momentos. Ao final do experimento, a obesidade culminou em maior velocidade de encurtamento da parede posterior do ventrículo esquerdo (VEPP), como observado nos grupos OB e OL. Considerando-se o desempenho diastólico, o grupo OL apresentou menor relação E/A do que o CL na 20ª semana. O Doppler tecidual da velocidade diastólica tardia do anel valvar mitral (A’ média) foi menor no CL do que no CO; S média e E’ média foram ampliadas da 16ª à 20ª semana no grupo OL.

**Tabela 2:** – Média e desvio-padrão de pressão arterial sistólica, medidas de estrutura e desempenho funcional do coração analisado por ecocardiograma e Doppler tecidual do ventrículo esquerdo, segundo grupo e momento de avaliação

Variável	Momento	Grupo			

		CO	OB	CL	OL
**PAS (mmHg)**	**16ª Sem**	119,4 ± 9,2	133,3 ± 12,3 *	119,5 ± 9,4	132,0 ± 9,6 ^‡^
	**20ª Sem**	129,6 ± 9,3	139,3 ± 12,6	103,0 ± 13,2 * ^§^	107,7 ± 7,4 ^† §^
**FC (bpm)**	**16ª Sem**	277 ± 41	272 ± 27	276 ± 48	273 ± 44
	**20ª Sem**	285 ± 32	266 ± 39	265 ± 39	277 ± 39
**AE (mm)**	**16ª Sem**	5,47 ± 0,79	5,80 ± 0,60	5,87 ± 0,74	5,81 ± 0,89
	**20ª Sem**	5,69 ± 0,56	5,95 ± 0,55	5,70 ± 0,60	5,77 ± 0,67
**AE/AO**	**16ª Sem**	1,37 ± 0,18	1,45 ± 0,14	1,48 ± 0,14	1,42 ± 0,18
	**20ª Sem**	1,42 ± 0,16	1,44 ± 0,10	1,40 ± 0,11	1,42 ± 0,12
**EDPP (mm)**	**16ª Sem**	1,317 ± 0,072	1,374 ± 0,044	1,313 ± 0,071	1,361 ± 0,058
	**20ª Sem**	1,272 ± 0,067 ^§^	1,305 ± 0,043 ^§^	1,262 ± 0,085 ^§^	1,271 ± 0,061 ^§^
**DDVE (mm)**	**16ª Sem**	7,95 ± 0,64	7,91 ± 0,37	7,80 ± 0,57	7,82 ± 0,44
	**20ª Sem**	8,11 ± 0,41	8,15 ± 0,26	8,06 ± 0,54	8,08 ± 0,52
**DDVE/ Tíbia (mm/mm)**	**16ª Sem**	0,184 ± 0,015	0,180 ± 0,008	0,182 ± 0,012	0,180 ± 0,007
	**20ª Sem**	0,188 ± 0,010	0,186 ± 0,007	0,188 ± 0,011	0,187 ± 0,012
**DSVE (mm)**	**16ª Sem**	3,65 ± 0,68	3,56 ± 0,39	3,54 ± 0,65	3,69 ± 0,56
	**20ª Sem**	3,66 ± 0,42	3,55 ± 0,50	3,86 ± 0,67	3,75 ± 0,62
**VEPP (mm/s)**	**16ª Sem**	40,44 ± 4,70	43,63 ± 2,95	42,31 ± 5,11	39,29 ± 3,96
	**20ª Sem**	42,92 ± 4,45	48,72 ± 4,81 * ^§^	42,82 ± 3,60	47,96 ± 4,03 ^‡ §^
**FE**	**16ª Sem**	0,900 ± 0,039	0,907 ± 0,023	0,903 ± 0,037	0,892 ± 0,037
	**20ª Sem**	0,907 ± 0,022	0,914 ± 0,033	0,887 ± 0,039	0,898 ± 0,037
**EE (%)**	**16ª Sem**	54,32 ± 6,38	55,00 ± 3,72	54,83 ± 6,28	52,92 ± 5,41
	**20ª Sem**	54,94 ± 3,58	56,49 ± 5,67	52,34 ± 5,73	53,83 ± 5,45
**E/A**	**16ª Sem**	1,65 ± 0,35	1,49 ± 0,25	1,52 ± 0,25	1,43 ± 0,23
	**20ª Sem**	1,60 ± 0,33	1,50 ± 0,23	1,74 ± 0,27	1,39 ± 0,26 ^‡^
**TDE (ms)**	**16ª Sem**	50,09 ± 6,85	49,50 ± 4,56	47,64 ± 8,69	51,10 ± 6,19
	**20ª Sem**	47,64 ± 7,47	50,58 ± 6,59	50,17 ± 5,84	54,40 ± 5,77
**TRIVn**	**16ª Sem**	58,88 ± 6,98	58,12 ± 4,22	55,38 ± 7,72	54,66 ± 5,26
	**20ª Sem**	53,60 ± 4,22	52,47 ± 4,87	53,49 ± 7,17	52,72 ± 3,78
**S’ média (cm/s)**	**16ª Sem**	3,57 ± 0,31	3,79 ± 0,28	3,72 ± 0,24	3,79 ± 0,45
	**20ª Sem**	4,00 ± 0,24 §	4,05 ± 0,47	3,91 ± 0,29	4,19 ± 0,27 ^§^
**E’ média (cm/s)**	**16ª Sem**	4,62 ± 0,53	4,23 ± 0,40	4,25 ± 0,39	4,04 ± 0,53
	**20ª Sem**	4,85 ± 0,57	4,80 ± 0,32 ^§^	4,83 ± 0,38 ^§^	4,92 ± 0,52 ^§^
**A’ média (cm/s)**	**16ª Sem**	3,75 ± 0,86	3,85 ± 0,59	3,78 ± 0,83	3,49 ± 0,50
	**20ª Sem**	4,37 ± 0,87	3,78 ± 1,08	3,61 ± 0,75 *	4,31 ± 0,81 ^§^
**E/E’**	**16ª Sem**	16,80 ± 3,62	18,76 ± 3,13	18,12 ± 2,27	18,86 ± 2,61
	**20ª Sem**	17,89 ± 2,59	18,79 ± 2,35	17,27 ± 1,52	17,22 ± 2,51

PAS: pressão arterial sistólica; FC: frequência cardíaca; AE/AO: relação entre os diâmetros do átrio esquerdo (AE) e da artéria aorta (AO); EDPP: espessura diastólica da parede posterior; DDVE: diâmetro diastólico do ventrículo esquerdo; DSVE: diâmetro sistólico do ventrículo esquerdo; VEPP: velocidade de encurtamento da parede posterior do ventrículo esquerdo; FE: fração de ejeção; EE: encurtamento endocárdico; E/A: relação entre as ondas E e A do fluxo transmitral; TRIVn: tempo de relaxamento isovolumétrico normalizado pela frequência cardíaca (R-R0,5); TDE: tempo de desaceleração da onda E; S’: velocidade sistólica do anel valvar mitral ao Doppler tecidual (TDI); E’: TDI da velocidade diastólica do anel valvar mitral (média entre paredes septal e lateral); A’: TDI da velocidade diastólica tardia do anel valvar mitral (média das paredes septal e lateral); E/E’: relação obtida entre as velocidades de fluxo inicial transvalvar mitral e do TDI do anel valvar mitral; CO: grupo Controle; OB: grupo Obeso; CL: grupo controle sob losartan; OL: grupo obeso sob losartan. Efeitos de grupo: * p<0,05 comparado ao CO; † p<0,05 comparado ao OB; ‡ p<0,05 comparado ao CL; Efeito de momento: § p<0,05 comparado à 16ª semana (Sem); Two-Way RM ANOVA e teste de Bonferroni.

**Figura 1:**
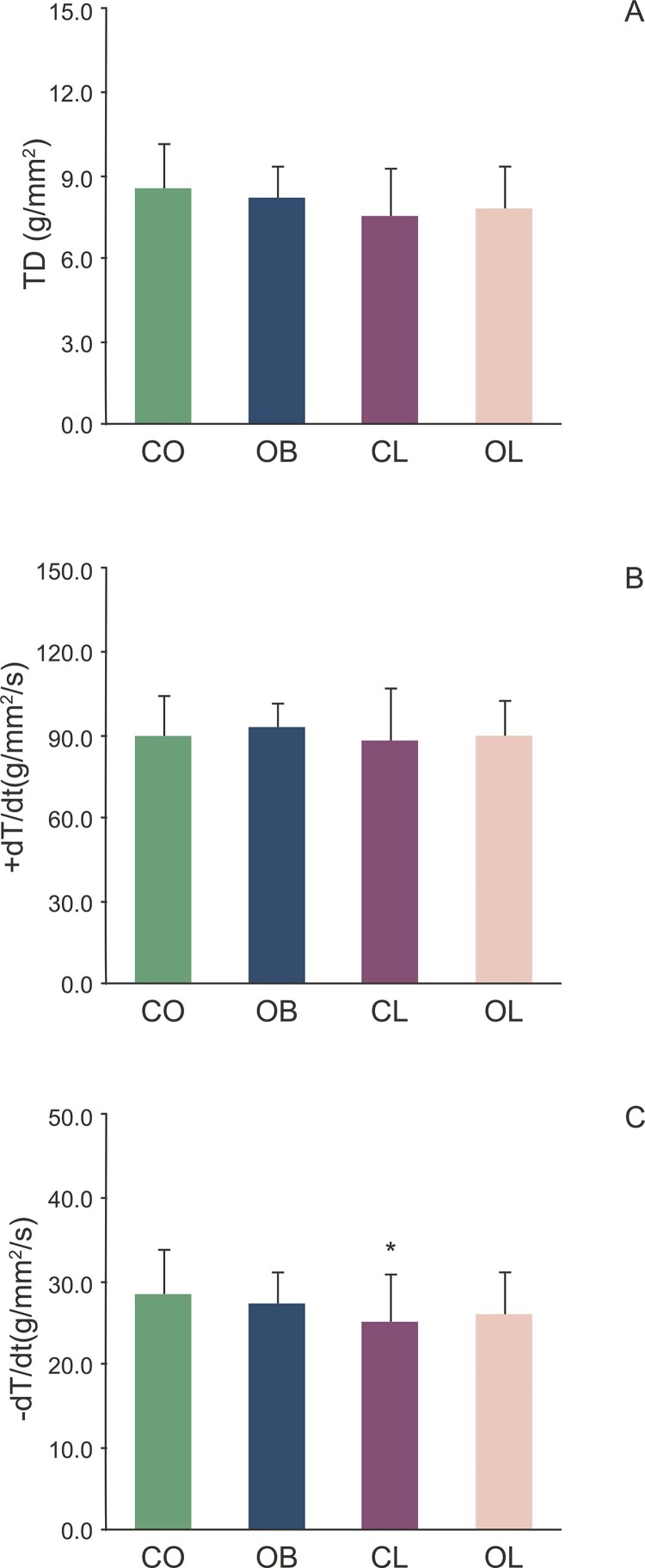
– Avaliação funcional do músculo papilar em situação basal com concentração extracelular de cálcio de 2,5 mM; valores em média±desvio-padrão; (A) TD: tensão máxima desenvolvida; (B) +dT/dt: velocidade máxima de variação da TD; (C) –dT/dt: velocidade máxima de variação de decréscimo da TD; CO: grupo Controle; OB: grupo Obeso; CL: grupo Controle sob losartan; OL: grupo Obeso sob losartan. * p<0,05 comparado ao CO; Two-Way ANOVA e teste de Tukey.

**Figura 2:**
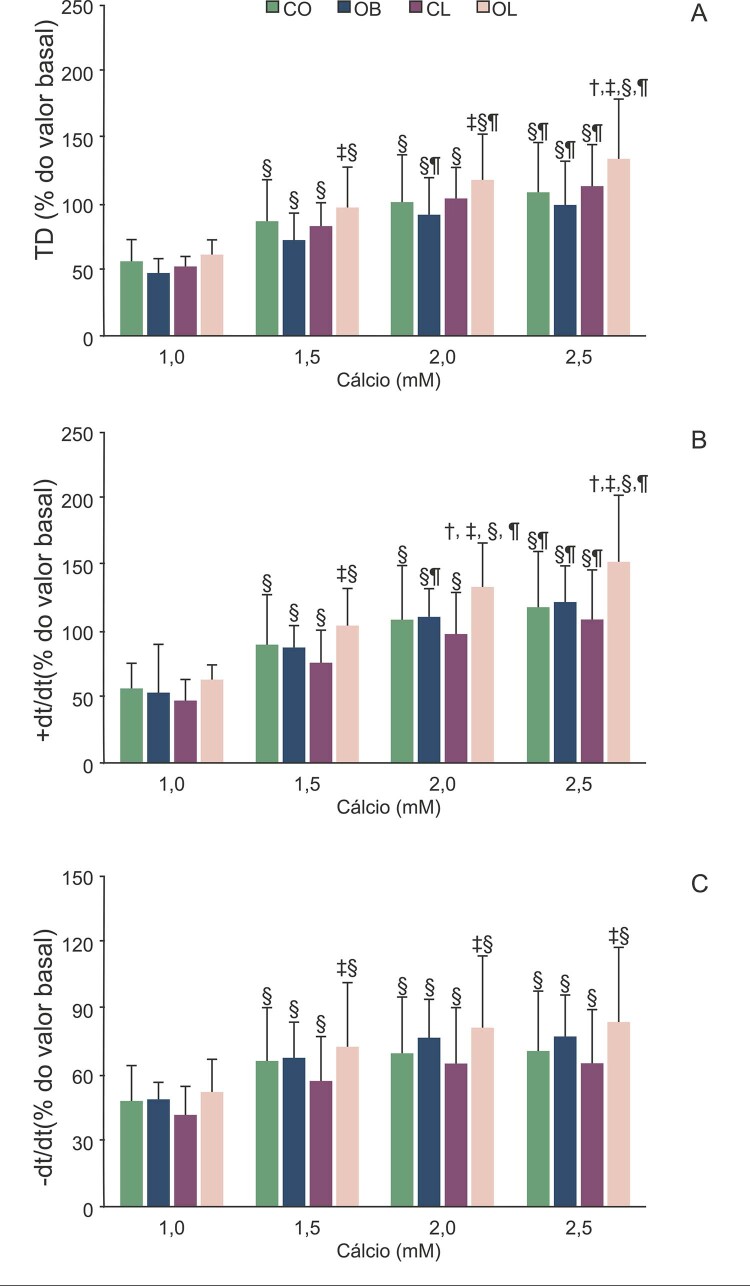
– *Avaliação funcional do músculo papilar segundo concentração extracelular de cálcio (1,0-2,5 mM). Resultados expressos em relação ao valor basal com concentração extracelular de cálcio de 0,5 mM; (média±desvio-padrão); (A) TD: tensão máxima desenvolvida; (B) +dT/dt: velocidade máxima de variação positiva da TD; (C) -dT/dt: velocidade máxima de decréscimo da TD. CO: grupo Controle; CL: grupo Controle sob losartan; OB: grupo Obeso; OL: grupo Obeso sob losartan. Efeito de grupo: † p<0,05 vs OB; ‡ p<0,05 vs CL. Efeito de Cálcio: §, p<0,05 vs 1,0 mM; ¶, p<0,05 vs 1,5 mM;Two-Way RM ANOVA e teste de Bonferroni.*

O desempenho funcional dos músculos papilares é mostrado nas Figuras 1 a 4 . Em condições basais, os índices de TD e +dt/dt foram similares entre os grupos ( Figura 1A e 1B ), enquanto a -dt/dt foi menor no CL do que no CO ( Figura 1C ). A influência da elevação das concentrações de cálcio sobre a função dos músculos papilares é apresentada na Figura 2 . O aumento da [Ca^2^]_0_ de 1,0 a 2,5 mM resultou em resposta progressivamente maior da TD, +dt/dt e -dT/dt em todos os grupos. Nas concentrações de cálcio de 1,5, 2,0 e 2,5 mM, o OL mostrou os valores superiores de TD, +dt/dt e -dT/dt em comparação ao CL. Na manobra de 2,5 mM de [Ca^2^]_0_, as variáveis TD (CO, 109±37; OB, 113±31; CL, 98±33; OL, 134±46 %) e +dt/dt (CO, 118±43; OB, 122±27; CL, 109±37; OL, 153±49 %) foram maiores no OL do que no OB ( Figura 2A e 2B ).

**Figura 3:**
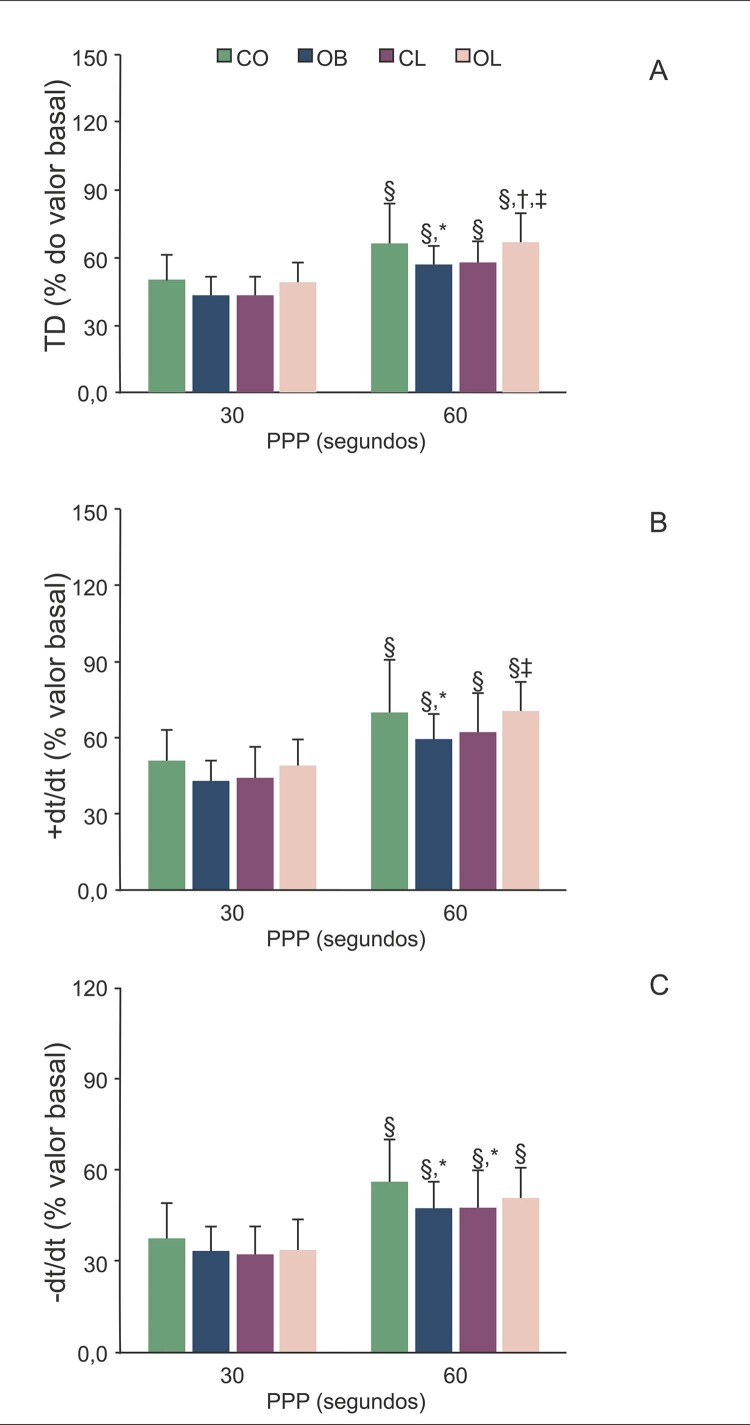
– Avaliação do músculo papilar isolado, segundo tempo de potencial pós-pausa (PPP). Resultados expressos em relação ao valor basal com concentração extracelular de cálcio de 0,5 mM (média±desvio-padrão); (A) TD: tensão máxima desenvolvida; (B) +dT/dt: velocidade máxima de variação positiva da TD; (C) -dT/dt: velocidade máxima de decréscimo da TD. CO: grupo Controle; CL: grupo Controle sob losartan; OB: grupo Obeso; OL: grupo Obeso sob losartan. Efeito de PPP: §, p<0,05 vs 30s; Efeito de grupo: * p<0,05 vs CO; † p<0,05 vs OB; ‡ p<0,05 vs CL. Two-Way RM ANOVA e teste de Bonferroni.

Na Figura 3 , são apresentados os resultados de desempenho funcional de músculos papilares em resposta ao PPP. A variação do PPP de 30 a 60s culminou em aumento dos valores de TD, +dt/dt e -dt/dt, em geral. No PPP de 60s, o grupo OB mostrou menores medidas de TD, +dt/dt e -dt/dt do que CO; o OL revelou maior TD (C, 65,7±23,6; OB, 56,3±13,9; CL, 58,0±17,4; OL, 66,4±17,4 %) do que os grupos OB e CL e valores superiores de +dt/dt (C, 70,0±25,4; OB, 59,3±15,9; CL, 62,7±20,0; OL, 70,7±20,7%) em relação ao CL.

**Figura 4:**
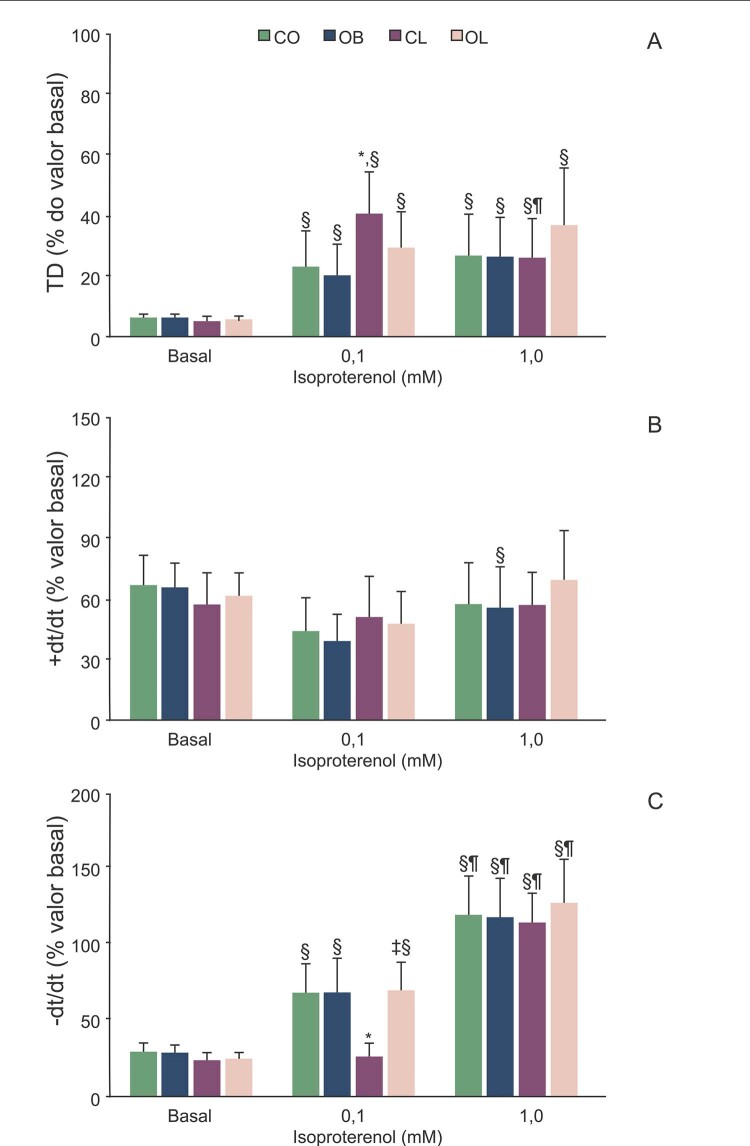
– Avaliação do músculo papilar isolado, segundo estímulo com isoproterenol (0,1 e 1,0 mM). Resultados expressos em relação ao valor basal com concentração extracelular de cálcio de 1,0 mM (média±desvio-padrão); (A) TD: tensão máxima desenvolvida; (B) +dT/dt: velocidade máxima de variação positiva da TD; (C) -dT/dt: velocidade máxima de decréscimo da TD. CO: grupo Controle; CL: grupo Controle sob losartan; OB: grupo Obeso; OL: grupo Obeso sob losartan. Efeito de Isoproterenol: §, p<0,05 vs Basal; ¶, p<0,05 vs 0,1 mM; Efeito de grupo: * p<0,05 vs CO; † p<0,05 vs OB; ‡ p<0,05 vs CL. Two-Way RM ANOVA e teste de Bonferroni.

Sobre as manobras de estimulação β-adrenérgica ( Figura 4 ), as concentrações de 0,1 e 1mM repercutiram em aumento da TD em comparação às condições basais. A manobra de 1mM de isoproterenol resultou em redução da +dt/dt no OB ( Figura 4B ) e ampliou as medidas -dt/dt em todos os grupos, em comparação às concentrações de base e de 0,1mM ( Figura 4C ). Considerando-se o efeito de grupo, o CL mostrou maior TD (C, 22,8±11,4; OB, 19,5±10,9; CL, 40,4±13,6; OL, 28,7±11,9 %) e menor -dt/dt do que o C (C, 67,5±18,5; OB, 67,2±22,6; CL, 25,3±9,2; OL, 68,8±19,1 %), em resposta a 0,1mM de isoproterenol.

## Discussão

Este trabalho foi proposto para avaliar a influência do antagonismo de receptores AT_1_ sobre características cardiovasculares em ratos obesos por dieta. De fato, os ratos obesos exibiram alterações de PAS, hipertrofia do VE, mudanças no desempenho sistólico avaliado por meio de ecocardiograma e distúrbios na função do músculo papilar. Grande parte desses efeitos foi amenizada com a administração de losartan, um fármaco antagonista de receptores AT_1_.

O presente modelo experimental é caracterizado pela indução de obesidade a partir da administração de dieta hiperlipídica, com predomínio de ácidos graxos saturados.^9 , 16^ Nesse sentido, apesar do consumo calórico inalterado entre os grupos, os animais obesos mostraram maiores medidas de ingestão lipídica e eficiência energética em comparação aos respectivos controles. Como resultado, os valores de massa corporal e adiposidade foram também maiores na obesidade. Em razão da maior densidade energética dos lipídeos, o consumo de dietas hiperlipídicas se associa com acúmulo de reservas corporais e hipertrofia do tecido adiposo. Com isso, a variação ponderal positiva dos obesos resultou, possivelmente, de aumento da adiposidade, conforme previamente documentado.^9 , 11 , 19^

Após a 16ª semana de experimento, a PAS mostrou-se maior na obesidade. A associação entre obesidade e alterações pressóricas foi também comprovada por outros estudos.^8 , 11 , 17^ Além disso, a PAS foi cronicamente ampliada após estresse físico^32^ e ao longo do tempo,^8^ ainda que os níveis basais estivessem inalterados ao final do experimento. Em geral, fatores inflamatórios e/ou neuro-hormonais relacionados com o excesso de tecido adiposo contribuem para a ocorrência de desordens hemodinâmicas em obesos.^20 , 23^ Na vigência de losartan, os níveis de PAS foram reduzidos, confirmando a participação do SRAA na promoção de distúrbios hemodinâmicos pressóricos derivados da obesidade.

Por sua vez, persistente aumento na PAS tem sido associado com maior pós-carga, deformação parietal e hipertrofia cardíaca.^33 , 34^ Nossos resultados confirmaram hipertrofia ventricular e elevado desempenho sistólico, comprovado pela maior VEPP na obesidade ( Tabela 2 ). A função sistólica é afetada por diferentes fatores, incluindo-se frequência cardíaca, contratilidade, além de mudanças na pré e pós-carga.^33^ Embora a obesidade não tenha modificado a frequência cardíaca e a geometria ventricular, maiores medidas de parede poderiam preservar, ou ainda, diminuir a pré-carga. Nesse estado, entretanto, a reduzida pré-carga poderia causar menor ejeção,^11 , 33^ o que não foi confirmado pelos resultados. Provavelmente, o maior desempenho sistólico se associa com hipertrofia ventricular e/ou mudanças na pós-carga no grupo OB. Cabe dizer que a pós-carga é uma variável mecânica diretamente influenciada por alterações na pressão e no diâmetro intraventricular e inversamente relacionada com a espessura da parede ventricular.^33 , 34^

Entretanto, a avaliação da função papilar mostrou que a obesidade, *per se* , não se associou com alterações basais, nem em resposta a diferentes concentrações de Ca^2^ e isoproterenol. Estudo prévio mostrou diminuição da força contrátil e outros distúrbios funcionais em condições basais de músculos papilares de obesos.^35^ Lima-Leopoldo et al.^7^ mostraram que a elevação da concentração extracelular de Ca^2^ resultou em menor resposta nos índices de função miocárdica na contração (TD) e no relaxamento (-dT/dt) na obesidade. Essas divergências podem ter relação com diferenças na composição das dietas, incluindo-se acréscimo de açúcar^7^ e/ou perfil lipídico constituinte das formulações. Utilizando modelo similar ao do presente trabalho, Vileigas et al.^16^ também constataram ausência de diferenças na função miocárdica em preparação de músculo papilar na condição basal e após acréscimo de isoproterenol.

Quanto à avaliação de potencial pós-pausa (PPP), pode-se afirmar que a obesidade promoveu disfunção miocárdica, muito provavelmente, em razão de alterações no trânsito intracelular de Ca^2^. A manobra de 60s reduziu a TD, +dT/dt e -dT/dt no miocárdio de ratos obesos ( Figura 3 ). Os dados são consistentes com estudo prévio que mostrou menor resposta contrátil em ratos obesos *Zucker* após 60s de PPP.^35^ Como -dT/dt é influenciada pela frequência de absorção de íons cálcio pelo retículo sarcoplasmático,^7^ a menor recaptura de Ca^2^ demonstrada pela -dT/dt nos ratos obesos sugere que a atividade da proteína SERCA2 foi deprimida. A diminuição da -dT/dt com altas concentrações citosólicas de Ca^2^ sugere que a ativação da SERCA2 via Ca^2^ - *calmodulina cinase* pode ser deprimida pela obesidade. A redução significativa da TD nos ratos obesos poderia ser resultante de redução de Ca^2^no retículo sarcoplasmático e também de menor liberação de Ca^2^ por meio dos receptores *rianodínicos* .

Provavelmente, os distúrbios no trânsito Ca^2^ e na contratilidade miocárdica em obesos decorrem da estimulação do SRAA. Quando comparados aos grupos OB e CL, os animais do OL mostraram melhor desempenho contrátil em resposta às manobras de elevação de Ca^2^, PPP e isoproterenol ( Figuras 2-4 ). Considerando-se a manutenção de elevada VEPP e o comportamento mecânico do músculo papilar na vigência de losartan, é provável que o desempenho sistólico tenha sido regulado por maior sensibilidade ao Ca^2^ no grupo OL. Nessa perspectiva, não se pode descartar um possível efeito metabólico do bloqueio de AT_1_, propiciando maior eficiência energética a partir da combustão melhorada de macronutrientes, em especial, lipídeos.^24 , 36^ Excessiva oferta de ácidos graxos pode culminar em maior atividade mitocondrial, estimulando mecanismos relacionados com aumento no trânsito de Ca^2^.^19 , 23^ Em experimento prévio, intervenção com losartan resultou na inibição de mecanismos moleculares de resistência à insulina no miocárdio, normalizando também o desempenho contrátil do coração em ratos obesos por dieta de cafeteria.^11^ Recentemente, o bloqueio de AT_1_ culminou em melhora na função mitocondrial em ratos obesos com resistência à insulina.^37^

Nessa perspectiva, as repercussões clínicas dos achados deste estudo são variadas. Condições de ativação do SRAA têm sido associadas a desordens metabólicas e cardiopatias.^23^ No presente trabalho, tem-se a descrição de distúrbios de resposta contrátil que possibilitam a proposição de intervenções voltadas ao tratamento cardiovascular em obesos.

Não obstante, embora esses efeitos tenham sido amenizados com antagonismo de AT_1_, não está descartada a participação de outras variáveis, comuns à dieta hiperlipídica de forma isolada, como causa de remodelação cardíaca. Em estudo prévio,^8^ mostramos que o consumo aumentado de lipídeos tem relação direta com indicadores de resposta cardiovascular na obesidade. Nesse sentido, essa é uma importante limitação do trabalho e novas investigações devem ser desenvolvidas para melhor esclarecer o papel isolado de ácidos graxos saturados e insaturados no presente modelo experimental.

## Conclusão

Os resultados permitem concluir que a obesidade induzida por dieta promove remodelação cardíaca, sustentada por hipertrofia ventricular e disfunção miocárdica. Considerando-se que o Losartan amenizou grande parte dessas desordens, confirma-se a hipótese inicial desta investigação, segundo a qual a estimulação de receptores AT_1_ está associada com prejuízos na função miocárdica em ratos obesos.
