# Supplementary material for: Bloqueio de Receptores AT1 Melhora o Desempenho Funcional Miocárdico na Obesidade
Source: Arq Bras Cardiol. 2020 Jul 28;115(1):17–28. [Article in Portuguese] doi: 10.36660/abc.20190131 (PMC8384332; doi:10.36660/abc.20190131)
Supplement: Translation

# Translation: AT_1_Receptor Blockade Improves Myocardial Functional Performance in Obesity

**Authors:** Silvio Assis de Oliveira-Junior, Nayara de Araújo Muzili, Marianna Rabelo de Carvalho, Gabriel Elias Ota, Camila Souza de Morais, Larissa Fregapani da Costa Vieira, Mateus Oliveira Ortiz, Dijon H. S. Campos, Marcelo Diacardia Mariano Cezar, Marina Politi Okoshi, Katashi Okoshi, Antonio C. Cicogna, Paula Felippe Martinez

**Affiliations:** 1 Brazil Universidade Federal de Mato Grosso do Sul, Campo Grande, MS – Brazil; 2 Brazil Faculdade de Medicina de Botucatu – UNESP, Botucatu, SP - Brazil; 3 Brazil Faculdade de Ciências Sociais e Agrárias de Itapeva (FAIT), Itapeva, SP - Brazil; 4 Brazil Universidade Estadual de São Paulo (UNESP), Departamento de Medicina Interna, Botucatu, SP - Brazil; 5 Brazil Universidade Estadual Paulista Júlio de Mesquita Filho - Faculdade de Medicina - Campus de Botucatu, Botucatu, SP - Brazil

**Keywords:** Cardiovascular Diseases, Obesity, Losartan/therapeutic use, Angiotensin II Type 1 Receptor Blockers/therapeutic use, Rats, Diet, High-fat/methods

## Abstract

**Background:**

Obesity has been associated with chronic activation of the renin-angiotensin-aldosterone system and with significant changes in cardiac performance.

**Objective:**

To assess the impact of a blockade of angiotensin-II receptor type 1 (AT_1_receptor) on morphology and on myocardial functional performance in rats with high-fat diet- induced obesity.

**Methods:**

Wistar rats (n=48) were submitted to control (2.9 kcal/g) or high-fat (3.6 kcal/g) diet for 20 weeks. After the 16^th^week they were divided into four groups: Control (CO), Obese (OB), Control Losartan (CL) and Obese Losartan (OL). CL and OL received losartan (30 mg/kg/day) in drinking water for four weeks. Subsequently, body composition, systolic blood pressure (SBP) and echocardiographic variables were analyzed. Papillary muscle function was assessed at baseline with 2.50 mM calcium concentration ([Ca^2+^]_o_) and after inotropic maneuvers: post-pause potentiation (PPP), [Ca^2+^]_o_elevation, and during beta-adrenergic stimulation with isoproterenol. Analysis of the results was performed by the Two-Way ANOVA and by the appropriate comparison test. The level of significance was set at 5%.

**Results:**

Although SBP change had been not maintained at the end of the experiment, obesity was associated with cardiac hypertrophy and with increased left ventricle posterior wall shortening velocity. In the study of papillary muscles in basal condition, CL showed lower developed tension maximum negative variation velocity (-dT/dt) than CO. The 60s PPP promoted lower -dT/dt and maximum developed tension (DT) in OB and CL compared with CO, and higher relative DT variation and maximum positive variation velocity (+dT/dt) in OL compared with CL and OB. Under 1.5, 2.0, and 2.5mM [Ca^2+^]_o_, the OL group showed higher -dT/dt than CL.

**Conclusion:**

Losartan improves myocardial function in high-fat diet-induced obesity. (Arq Bras Cardiol. 2020;115(1):17-28)

## Introduction

Obesity is a chronic and multifactorial disease resulting from interaction among many etiological factors.^1 , 2^ This disease is a nutritional and metabolic dysfunction that may be associated with dyslipidemia, insulin resistance and cardiovascular diseases.^3^ Clinical studies have shown obesity may cause morphological and functional changes in the heart.^4 , 5^ Moreover, experimental research proved this condition is associated with myocardial hypertrophy,^6 - 8^ interstitial fibrosis,^8 , 9^ and several molecular changes.^10 , 11^ These responses include disorders in expression and functioning of peptides involved with intracellular calcium handling during muscle contraction and relaxation.^7 , 12 - 14^

However, there are important divergences among studies regarding potential effects of high-fat diet induced obesity on myocardial performance. Jacobsen et al.^15^ found increased contractile phase during inotropic maneuver of papillary muscle in obese rats after three weeks of diet; other authors have found higher myocardial shortening velocity in experiments of 20,^8^ 30,^11^ 33,^13^ and 35 weeks.^16^ Other investigations have reported impaired cardiac contraction, showed by *in vitro* papillary muscles analysis of obese rats in experimental models with15 weeks of diet.^7 , 17 , 18^ There are also reports of unchanged cardiac function after 20,^9^ 30,^19^ and 32^14^ weeks of dietary intervention. Therefore, cardiac performance should be further studied in high-fat diet-induced obesity experiments.

Obesity regards greater activity of the renin-angiotensin-aldosterone system (RAAS).^11 , 20 , 21^ High levels of angiotensin-II (Ang-II) coupling to receptors type I (AT_1_) exert a vasoconstrictor and a trophic effect on myocardium, stimulating several intracellular signaling cascades and multiple physiological responses.^21 - 23^ RAAS activation is the main mechanism responsible for blood pressure disorders and cardiac remodeling in obesity; these effects were attenuated after AT_1_antagonism.^16 , 11 , 21 , 24^ However, when considering the *in vitro* analysis of the papillary muscle, the association between RAAS activation and ventricular remodeling in obesity models based on high-fat diet administration is scarcely studied.

The *in vitro* preparation of papillary muscle allows myocardial contractile capacity measurements in terms of shortening and force generation, despite changes in load, heart rate and heart geometry; such conditions modify mechanical performance *in vivo* .^7 , 13 , 17 , 19^ Using inotropic and lusitropic maneuvers, myocardial performance may also be studied to identify changes in contraction and relaxation that could not be observed under baseline conditions. The most used maneuvers are post-pause potentiation, extracellular [Ca^2^] elevation and beta-adrenergic stimulation.^7^

From this perspective, the objective of this study was to assess the influence of AT_1_blockade on cardiac morphology and performance using *in vitro* papillary muscle analysis in rats with saturated high-fat diet induced obesity. The initial hypothesis is that obesity is associated with changes in myocardial functional performance, sustained under different stimulation conditions; these responses are attenuated by AT_1_receptor antagonism.

## Methods

### Animal and experimental design

Male Wistar rats (n=48), aged 30 days-old were used from the Animal Center of São Paulo State University – UNESP – Botucatu/SP, Brazil. The sample size definition was based on a previous study,^19^ developed with a similar experimental model and functional analysis of the isolated papillary muscle.

Firstly, animals were divided into two groups: control (CO), treated with control diet (2.9 kcal/g), and obese (OB), fed with high-fat diet with a predominance of saturated fatty acids (3.6 kcal/g).^9^ The following ingredients were used for both dietary preparations: corn bran, soybean bran and hulls, dextrin, and palm and soybean oils, plus vitamin and mineral supplementation. In terms of saturated/unsaturated fatty acids content,^9 , 16^ while the control diet presented 61.6/38.4%, the high-fat diet showed 64.8/35.2%.

After 16 weeks, the animals were allocated into four groups: CO, OB, CL and OL. For another four weeks, while CO and OB continued to receive their respective diets, CL and OL also received losartan in drinking water (30 mg/kg/day).^11^ The animals were kept in individual cages at 22±2°C (room temperature), 55±5% humidity, and 12 hours light/dark lighting cycles. The experimental protocol was reviewed and approved by the Ethics Committee on Animal Experiments of the Botucatu Medical School (protocol 1000/2013).

### Cardiovascular study

The cardiovascular study involved systolic blood pressure (SBP) measurement, cardiac morphology assessment, echocardiographic functional analysis and *in vitro* papillary muscle study. SBP and echocardiogram analysis were performed at 16 and 20 weeks of the experiment. SBP was obtained by plethysmography^26^ using a sphygmomanometer (Narco Bio-Systems®, model 709-0610 - International Biomedical, Austin, TX, USA). For echocardiography, the animals were anesthetized with a mixture of ketamine hydrochloride (50 mg/kg) and xylidine hydrochloride (1 mg/kg) administered intramuscularly. After trichotomy in the anterior thorax, each animal was positioned in the left lateral position. For cardiac geometry analysis, one-dimensional images (M-mode) were obtained with the ultrasound beam adjusted in the two-dimensional mode, keeping the transducer on the parasternal position and smaller axis.

Left ventricle (LV) imaging was obtained by positioning the M-mode cursor below the mitral valve plane at the papillary muscles level.^27^ Aortic and left atrial images were obtained with the M-mode cursor positioned at the aortic plane level. Images were recorded on a printer (model UP-890, Sony Co.). Cardiac structures were measured manually with a caliper. During the maximum ventricular cavity diameter, LV diastolic diameter (LVDD), LV posterior wall diastolic thickness (LVDT), and interventricular septum (IVDT) were measured. The LV systolic diameter (LVSD) was assessed during the minimum cavity diameter. The left atrium (LA) was measured at its maximum diameter. LV weight (LVW) was estimated according to the following formula: LVW = [(LVDD+LVDT+IVDT)^3^- (LVDD)^3^] x 1.04. The ratio between LVDD and tibia length was also considered.

LV systolic function was assessed by posterior wall shortening velocity (PWSV) and percentage of eendocardium fractional shortening (% ES) = [(LVDD-LVSD)/LVDD]. The diastolic function was analyzed by the following indexes: 1) ratio between the initial filling flow velocity peaks (E wave) and the atrial contraction (A wave) of the transmitral flow (E/A); 2) E wave deceleration time (EDT); 3) isovolumetric relaxation time (IVRT); 4) early mitral annulus diastolic displacement velocity peak (E’) and late mitral annulus diastolic displacement velocity peak (A’) obtained by tissue Doppler; and 5) ratio between the waves E and E’ (E/E’). All measurements were performed by the same expert according to the American Society of Echocardiography^28^ procedures, using an echocardiograph (General Electric Medical Systems, Vivid S6, Tirat Carmel, Israel), equipped with a multifrequency electronic transducer (5-11.5 MHz).

### General characterization and *in vitro* analysis of myocardial performance

Caloric intake was assessed daily.^6 - 8^ Feeding efficiency was obtained from the relationship between body weight variation and total energy intake.^6 - 8^ Body weight was measured weekly, while weight gain was obtained from the difference between initial and final body weight values. Adipose tissue from the retroperitoneal, epididymal, and visceral regions was used to determine body fat content.^6 - 12^

Myocardial performance was assessed by *in vitro* study with papillary muscle isolated from LV.^7 , 16 , 18 , 29^ After 20 weeks, animals were submitted to intraperitoneal anesthesia with ketamine hydrochloride (80 mg/kg), xylazine (5 mg/kg), and euthanasia. After median thoracotomy, the heart was removed and dissected. Atria, right ventricle (RVW) as well as left ventricle (LVW) were weighted for macroscopic morphological analysis. Dissected LV papillary muscles were placed between two stainless steel rings and positioned vertically within a glass chamber containing Krebs-Henseleit solution at 28°C, continuously oxygenated with O_2_(95%) and CO_2_(5%). The Krebs solution composition was the following: 118.5 mM NaCl; 4.69 mM KCl; 2.50 mM CaCl_2_; 1.16 mM MgSO_4_; 1.18 mM KH2PO_4_; 5.50 mM glucose; and 24.88 mM NaCO_3_. The lower end of the inferior ring was coupled to a 120T-20B force transducer (Kyowa, Tokyo, Japan) by a steel wire (1/15,000) running through a mercury-filled slot in the glass chamber floor.^7 , 16 , 18 , 29^

The muscles were kept on isotonic contraction against a light loading for 60 minutes; afterwards, they were then kept on isometric contraction and gradually stretched until the maximum developed tension (DT) was achieved. After 5 minutes under isotonic contraction, the muscles were placed back in isometric contraction to determine the tension-length curve (L_max_) peak. The papillary muscles behavior was assessed at baseline with a 2.50 mM calcium concentration ([Ca^2^]_o_) and after the following inotropic maneuvers: post-rest potentiation (PPP), extracellular [Ca^2^] elevation since 0.5 until 2.5 mM, and during beta-adrenergic stimulation with 0.1 and 1.0 mM isoproterenol. Post-pause potentiation was studied in extracellular [Ca^2^] equal to 1.50 mM, where the stimulus was stopped for 30 and 60 seconds before it restarted.^7 , 30^

After PPP, the papillary muscle response was assessed after extracellular [Ca^2^]_o_maneuver.^31^ Isometric contractile parameters were recorded after 10 minutes with progressive calcium addition (0.5 to 2.5mM) in the extracellular solution. The beta-adrenergic system stimulation has also been studied to test beta-adrenergic complex integrity, troponin C sensitivity, and calcium absorption by the sarcoplasmic reticulum.^7 , 31^ Beta adrenergic receptor stimulation was induced using cumulative isoproterenol concentrations (0.1 to 1.0 mM) in the presence of 1.0 mM [Ca^2^]_o_.

### Mechanical variables

Conventional mechanical responses at L_max_were obtained in isometric contraction: maximum developed tension normalized by the transverse sectional area of the papillary muscle (DT [g/mm^2^]) and maximum positive variation velocities (+dT/dt [g/mm^2^/s]); and maximum negative variation velocity (-dT/dt [g/mm^2^/s]) of maximum developed tension (DT), normalized by the transverse sectional area of the papillary muscle. The measures used to characterize papillary muscle size included length (mm), muscle weight (mg) and transverse sectional area (TSA [mm^2^]). At the end of each experiment, L_max_was measured with the *Gaertner* catheter (Gaertner Scientific Corporation, Chicago, IL, USA), and the muscle portion between the steel rings was cut and weighed. TSA was obtained from a ratio between muscle weight and length, assuming uniformity and a specific 1.0 gravity.

### Statistical analysis

A Sigma-Stat version 3.5 software was used for data analysis. Firstly, the results were subjected to normality analysis by the Kolmogorov-Smirnov test. Since the variables had parametric distribution, measures were presented as mean and standard-deviation. Nutritional results, body composition, cardiac morphology and functional performance of the papillary muscle were analyzed using the two-way analysis of variance ( *Two-Way* ANOVA) and the Tukey’s multiple comparisons test. SBP and echocardiogram measurements were analyzed by *Two-Way* ANOVA in the repeated measures (RM) model, and Bonferroni multiple comparison test. The level of significance was set at 5%.

## Results

Results of nutritional profile, body composition and cardiac macroscopic morphology are shown in Table 1 . Although caloric intake was unchanged, OB and OL showed higher fat intake and feed efficiency than CO and CL, respectively. Obesity was characterized by higher measures of body weight and adiposity.

**Table 1:** – Mean and standard deviation of nutritional variables, murinometry and cardiac morphology according with group

Variable		Group			

		CO	OB	CL	OL
**Nutritional Profile**	**Body weight (g)**	451 ± 58	507 ± 64 *	456 ± 49	517 ± 50^‡^
	**Caloric intake (Kcal)**	81.9 ± 8.2	80.7 ± 7.5	80.3 ± 9.2	78.7 ± 7.9
	**Total intake of unsaturated lipids (g)**	122 ± 12	235 ± 22 *	120 ± 14	230 ± 23^‡^
	**Total intake of saturated lipids (g)**	196 ± 20	433 ± 40 *	193 ± 22	422 ± 43^‡^
	**Feed efficiency (%)**	26.82 ± 2.11	32.22 ± 4.38 *	28.43 ± 0.94	35.12 ± 4.78^‡^
	**Adiposity (%)**	3.48 ± 0.73	5.19 ± 1.47 *	3.61 ± 1.27	5.50 ± 1.48^‡^
**Cardiac Morphology**	**Atria (g)**	0.096 ± 0.015	0.113 ± 0.015 *	0.092 ± 0.009	0.100 ± 0.022^†^
	**Atria/ Tibia (mg/mm)**	2.22 ± 0.33	2.59 ± 0.35 *	2.15 ± 0.21	2.29 ± 0.46^†^
	**RVW (g)**	0.231 ± 0.029	0.241 ± 0.030	0.230 ± 0.039	0.245 ± 0.040
	**RVW/Tibia (mg/mm)**	5.36 ± 0.68	5.50 ± 0.70	5.34 ± 0.79	5.64 ± 0.86
	**LVW (g)**	0.844 ± 0.083	0.950 ± 0.099	0.800 ± 0.082 *	0.799 ± 0.087^†^
	**LVW/Tibia (mg/mm)**	19.6 ± 1.7	21.7 ± 2.4 *	18.7 ± 1.5	18.4 ± 1.7^†^

RVW: right ventricular weight; LVW: left ventricular weight; CO: Control group; OB: Obese group; CL: Control Losartan group; OL: Obese Losartan group Group’s effects: * p <0.05 compared to CO; † p <0.05 compared to OB; ‡ p <0.05 compared to CL; Two-Way ANOVA and Tukey test.

OB presented higher values of atria weight and respective relationships between atrial weight and LV weight with tibia length compared to CO regarding cardiac morphology. Losartan promoted lower atrial and LV measurements comparing OL with OB in absolute values and when normalized by tibia length, as shown in Table 1 .

Table 2 presents SBP results, structure and performance of the heart, assessed by echocardiography. After 16 weeks, obesity was associated with higher SBP; losartan led to SBP reduction in CL and OL at the end of the experiment. The ratio between left ventricular diastolic diameter (LVDD) and tibia length was similar among groups and between the moments. At the end of the experiment, obesity culminated in a higher posterior wall shortening velocity (PWSV), as observed in OB and OL. Considering the diastolic performance, OL presented lower E/A ratio than CL at the 20^th^week. Tissue Doppler of late diastolic mitral valve annular velocity (A’ average) was lower in CL than CO; S average and E’ average were increased from week 16 to week 20 in OL.

**Table 2:** – Mean and standard deviation of systolic blood pressure, measures of structure and functional performance of the heart analyzed by echocardiogram and left ventricular tissue Doppler, according to group and time of assessment

Variable	Moment	Group			

		CO	OB	CL	OL
**SBP (mmHg)**	**16th Wks**	119.4 ± 9.2	133.3 ± 12.3 *	119.5 ± 9.4	132.0 ± 9.6^‡^
	**20th Wks**	129.6 ± 9.3	139.3 ± 12.6	103.0 ± 13.2 *^§^	107.7 ± 7.4^† §^
**HR (bpm)**	**16th Wks**	277 ± 41	272 ± 27	276 ± 48	273 ± 44
	**20th Wks**	285 ± 32	266 ± 39	265 ± 39	277 ± 39
**LA (mm)**	**16th Wks**	5.47 ± 0.79	5.80 ± 0.60	5.87 ± 0.74	5.81 ± 0.89
	**20th Wks**	5.69 ± 0.56	5.95 ± 0.55	5.70 ± 0.60	5.77 ± 0.67
**LA/AO**	**16th Wks**	1.37 ± 0.18	1.45 ± 0.14	1.48 ± 0.14	1.42 ± 0.18
	**20th Wks**	1.42 ± 0.16	1.44 ± 0.10	1.40 ± 0.11	1.42 ± 0.12
**LVDT (mm)**	**16th Wks**	1.317 ± 0.072	1.374 ± 0.044	1.313 ± 0.071	1.361 ± 0.058
	**20th Wks**	1.272 ± 0.067^§^	1.305 ± 0.043^§^	1.262 ± 0.085^§^	1.271 ± 0.061^§^
**LVDD (mm)**	**16th Wks**	7.95 ± 0.64	7.91 ± 0.37	7.80 ± 0.57	7.82 ± 0.44
	**20th Wks**	8.11 ± 0.41	8.15 ± 0.26	8.06 ± 0.54	8.08 ± 0.52
**LVDD/ Tibia (mm/mm)**	**16th Wks**	0.184 ± 0.015	0.180 ± 0.008	0.182 ± 0.012	0.180 ± 0.007
	**20th Wks**	0.188 ± 0.010	0.186 ± 0.007	0.188 ± 0.011	0.187 ± 0.012
**LVSD (mm)**	**16th Wks**	3.65 ± 0.68	3.56 ± 0.39	3.54 ± 0.65	3.69 ± 0.56
	**20th Wks**	3.66 ± 0.42	3.55 ± 0.50	3.86 ± 0.67	3.75 ± 0.62
**PWSV (mm/s)**	**16th Wks**	40.44 ± 4.70	43.63 ± 2.95	42.31 ± 5.11	39.29 ± 3.96
	**20th Wks**	42.92 ± 4.45	48.72 ± 4.81 *^§^	42.82 ± 3.60	47.96 ± 4.03^‡ §^
**EF**	**16th Wks**	0.900 ± 0.039	0.907 ± 0.023	0.903 ± 0.037	0.892 ± 0.037
	**20th Wks**	0.907 ± 0.022	0.914 ± 0.033	0.887 ± 0.039	0.898 ± 0.037
**ES (%)**	**16th Wks**	54.32 ± 6.38	55.00 ± 3.72	54.83 ± 6.28	52.92 ± 5.41
	**20th Wks**	54.94 ± 3.58	56.49 ± 5.67	52.34 ± 5.73	53.83 ± 5.45
**E/A**	**16th Wks**	1.65 ± 0.35	1.49 ± 0.25	1.52 ± 0.25	1.43 ± 0.23
	**20th Wks**	1.60 ± 0.33	1.50 ± 0.23	1.74 ± 0.27	1.39 ± 0.26^‡^
**EDT (ms)**	**16th Wks**	50.09 ± 6.85	49.50 ± 4.56	47.64 ± 8.69	51.10 ± 6.19
	**20th Wks**	47.64 ± 7.47	50.58 ± 6.59	50.17 ± 5.84	54.40 ± 5.77
**IVRT**	**16th Wks**	58.88 ± 6.98	58.12 ± 4.22	55.38 ± 7.72	54.66 ± 5.26
	**20th Wks**	53.60 ± 4.22	52.47 ± 4.87	53.49 ± 7.17	52.72 ± 3.78
**S’(cm/s)**	**16th Wks**	3.57 ± 0.31	3.79 ± 0.28	3.72 ± 0.24	3.79 ± 0.45
	**20th Wks**	4.00 ± 0.24^§^	4.05 ± 0.47	3.91 ± 0.29	4.19 ± 0.27^§^
**E’ (cm/s)**	**16th Wks**	4.62 ± 0.53	4.23 ± 0.40	4.25 ± 0.39	4.04 ± 0.53
	**20th Wks**	4.85 ± 0.57	4.80 ± 0.32^§^	4.83 ± 0.38^§^	4.92 ± 0.52^§^
**A’ (cm/s)**	**16th Wks**	3.75 ± 0.86	3.85 ± 0.59	3.78 ± 0.83	3.49 ± 0.50
	**20th Wks**	4.37 ± 0.87	3.78 ± 1.08	3.61 ± 0.75 *	4.31 ± 0.81^§^
**E/E’**	**16th Wks**	16.80 ± 3.62	18.76 ± 3.13	18.12 ± 2.27	18.86 ± 2.61
	**20th Wks**	17.89 ± 2.59	18.79 ± 2.35	17.27 ± 1.52	17.22 ± 2.51

SBP: systolic blood pressure; HR: heart rate; LA / AO: relationship between the diameters of the left atrium (LA) and the aortic artery (AO); LVDT: diastolic thickness of the posterior wall; LVDD: left ventricular diastolic diameter; LVSD: left ventricular systolic diameter; PWSV: posterior wall shortening velocity; EF: ejection fraction; ES: endocardial shortening; E / A: relationship between the E and A waves of the transmitral flow; IVRT: isovolumetric relaxation time; EDT: E wave deceleration time; S ‘: systolic velocity of the mitral valve ring at tissue Doppler (TDI); E ‘: TDI of the diastolic velocity of the mitral valve ring (mean between septal and lateral walls); A ‘: TDI of the late diastolic velocity of the mitral valve ring (mean of the septal and lateral walls); E / E ‘: relation obtained between the velocities of the initial mitral valve flow and the TDI of the mitral valve ring; CO: Control group; OB: Obese group; CL: Control Losartan group; OL: Obese Losartan group. Group’s effects: * p <0.05 compared to CO; † p <0.05 compared to OB; ‡ p <0.05 compared to CL; Moment’s effect: § p <0.05 compared to the 16th week (Wks); Two-Way RM ANOVA and Bonferroni test.

The functional performance of the papillary muscles is shown in Figures 1 to 4 . Under basal conditions, the DT and +dT/dt indexes were similar among groups ( Figure 1A and 1B ), while the -dT/dt was lower in CL than CO ( Figure 1C ). The effects of diverse calcium concentrations on papillary muscle performance are shown in Figure 2 . Increasing [Ca^2^]_o_from 1.0 to 2.5 mM resulted in higher DT, +dT/dt and -dT/dt values in all groups. OL showed higher DT, +dT/dt and -dT/dt values compared to CL at calcium concentrations of 1.5, 2.0, and 2.5 mM. In the 2.5 mM [Ca^2^]_o_maneuver, DT (CO, 109±37; OB, 113±31; CL, 98±33; OL, 134±46%) and +dT/dt measures (CO, 118±43; OB, 122±27; CL, 109±37; OL, 153±49%) were higher in OL than OB ( Figure 2A and 2B ).

**Figure 1:** – Functional papillary muscle assessment at baseline with extracellular [Ca^2+^] equal to 2.5 mM; results in mean±SD; (A) DT: maximum developed tension; (B) +dT/dt: maximum DT variation speed; (C) –dT/dt: maximum DT decrease; CO: Control group; OB: Obese group; CL: Control Losartan group; OL: Obese Losartan group. * p<0.05 compared to CO; Two-Way ANOVA and Tukey Test.

**Figure 2:** – *Functional papillary muscle assessment according to extracellular calcium concentration (1.0-2.5 mM). Results expressed regarding the baseline with extracellular [Ca^2+^] equal to 0.5 mM value (mean±SD); (A) DT: maximum developed tension; (B) +dT/dt: maximum positive DT change; (C) -dT/dt, maximum DT decrease. CO: Control group; CL: Control Losartan group; OB: Obese group; OL: Obese Losartan group. Group’s effect: † p<0.05 compared to OB; ‡ p<0.05 compared to CL. Calcium’s Effect: §, p<0.05 compared to 1.0 mM; ¶, p<0.05 compared to 1.5 mM; Two-Way RM ANOVA and Bonferroni Test.*

Figure 3 presents results of papillary muscles functional performance in response to PPP. In general, the PPP variation from 30 to 60s culminated in increased DT, +dT/dt and -dT/dt values. In the 60s PPP, OB group showed lower DT, +dT/dt and -dT/dt measurements than CO; OL showed higher DT (CO, 65.7±23.6; OB, 56.3±13.9; CL, 58.0±17.4; OL, 66.4±17.4%) than OB and CL, and higher +dT/dt values (CO, 70.0±14.9; OB, 59.3±15.9; CL, 62.7±20.0; OL, 70.7±20.7%) when compared with CL.

**Figure 3:** – Isolated papillary muscle assessement, according to post-pause potentiation (PPP) time. Results are expressed regarding the baseline with extracellular [Ca^2+^] equal to 0.5 mM value (mean±SD); (A) DT: maximum developed tension; (B) +dT/dt: maximum positive DT change; (C) –dT/dt: maximum DT decrease; CO: Control group; CL: Control Losartan group; OB: Obese group; OL: Obese Losartan group. PPP’s effect: §, p<0.05 compared to 30s; Group’s effect: * p<0.05 vs CO; † p<0.05 compared to OB; ‡ p<0.05 compared to CL. Two-Way RM ANOVA and Bonferroni Test.

Regarding the β-adrenergic stimulation maneuvers, according to Figure 4 , concentrations of 0.1 and 1mM showed an increase in DT when compared to basal conditions. The 1mM isoproterenol maneuver resulted in reduced +dT/dt in OB ( Figure 4B ) and increased the -dT/dt measurements in all groups when compared to baseline and 0.1mM concentrations ( Figure 4C ). Considering the group effect, CL showed higher DT (CO, 22.8±11.4; OB, 19.5±10.9; CL, 40.4±13.6; OL, 28.7±11.9%) and lower -dT/dt than CO (CO, 67.5±18.5; OB, 67.2±22.6; CL, 25.3±9.2; OL, 68.8±19.1%) in response to 0.1mM isoproterenol.

**Figure 4:** – Functional assessment of the isolated papillary muscle, according to Isoproterenol concentration. Results expressed regarding the baseline with extracellular [Ca^2+^] equal to 1.0 mM value (mean±SD); (A) DT: maximum developed tension; (B) +dT/dt: maximum positive DT change; (C) -dT/dt: maximum DT decrease; CO: Control group; OB: Obese group; CL: Control Losartan group; OL: Obese group under Losartan. Group’s effect: * p<0.05 compared to C; ‡ p<0.05 compared to CL. Isoproterenol’s Effect: §, p<0.05 compared to Baseline; ¶, p<0.05 vs 0.1 mM; Two-Way RM ANOVA and Bonferroni Test.

## Discussion

This study aimed to assess potential effects of AT_1_receptor antagonism on cardiovascular characteristics in obese rats. Obese rats exhibited SBP changes, LV hypertrophy, alterations in systolic performance assessed by echocardiography, and disorders of papillary muscle function. Most of these effects have been attenuated by the losartan administration, an AT_1_receptor antagonist intervention.

This experimental model is characterized by the induction of obesity from the high-fat diet administration, with a predominance of saturated fatty acids.^9 , 16^ In this context, despite the unchanged caloric consumption between groups, the obese animals showed higher measures of lipid intake and energy efficiency when compared to the respective control counterparts. As a result, body weight and adiposity values were also higher in obesity. Due to higher energy density of lipids, consumption of high-fat diets is associated with accumulation of body reserves and adipose tissue hypertrophy^9 , 16 - 19^ . Probably, the positive weight variation of obese animals resulted from increased adiposity, as previously reported.^9 , 11 , 19^

SBP was higher in obese after 16 experimental weeks. The association between obesity and blood pressure changes has also been confirmed by other studies.^8 , 11 , 17^ Also, SBP was chronically increased after physical stress^32^ and in response to experimental period,^8^ even though baseline levels were unchanged at the end of the experiment. In general, inflammatory and/or neurohormonal factors regarding excess adipose tissue contribute to the occurrence of hemodynamic disorders in obese.^20 , 23^ In the presence of losartan, SBP levels were reduced, confirming the RAAS participation in promoting obesity-derived hemodynamic pressure disorders.

In turn, persistent increase in SBP has been associated with higher afterload, parietal deformation and cardiac hypertrophy.^33 , 34^ The results of this study confirmed ventricular hypertrophy and high systolic performance, as shown by the higher PWSV in obesity according to Table 2 . Systolic function is affected by several factors, including heart rate, contractility, and changes in preload and afterload.^33^ Although obesity did not change heart rate and ventricular geometry, larger wall measurements could preserve or decrease preload. However, reduced preload could cause lower ejection,^11 , 33^ which was not confirmed by the results. Likewise, increased systolic performance is associated with ventricular hypertrophy and/or changes in afterload in OB. The afterload is a mechanical variable directly influenced by changes in pressure and intraventricular diameter and inversely related to ventricular wall thickness.^33 , 34^

However, the papillary function assessment showed that obesity *per se* was not associated with basal changes, not only in response to various [Ca^2^] but also isoproterenol concentrations. A previous study showed decreased contractile strength and other functional disorders in basal conditions of obese papillary muscles.^35^ Lima-Leopoldo et al.^7^ showed that increased Ca^2^extracellular concentration resulted in lower values of myocardial parameters of contraction (DT) and relaxation (-dT/dt) in obesity. These divergences may regard differences in dietary compositions, including added sugar^7^ and/or lipid profile from formulations. Based on using a similar intervention to this study, Vileigas et al.^16^ also found unchanged myocardial function in papillary muscle preparation at baseline and after isoproterenol addition.

Regarding the PPP assessment, obesity promoted myocardialdysfunction, most probably due to changes in intracellular Ca^2^handling. The 60s maneuver reduced DT, +dT/dt and -dT/dt values in myocardium of obese rats, as in Figure 3 . The results agree with previous studies showing lower contractile response in obese Zucker rats after 60s of PPP^35^ . As -dT/dt is influenced by the frequency of calcium ions absorption into the sarcoplasmic reticulum,^7^ the lower Ca^2^recapture shown by -dT/dt in obese rats suggests that SERCA2 protein activity was reduced. Decreasing -dT/dt with high cytosolic Ca^2^concentrations suggests that activation of SERCA2 from Ca^2^/ *calmodulin-dependent protein kinase* may be shortened by obesity. Important reduction in DT of obese rats could result not only from Ca^2^reduction in the sarcoplasmic reticulum, but also from a lower Ca^2^release through the *Rianodine* receptors.

The disturbances in Ca^2^intracellular handling and myocardial contractility in obese rats probably result from the RAAS stimulation. When compared with OB and CL, OL animals showed better contractile performance in response to Ca^2^, PPP and isoproterenol elevation maneuvers ( Figures 2-4 ). Considering the high PWSV maintenance and the mechanical behavior of the papillary muscle in response to losartan, it is likely that systolic performance was regulated by greater sensitivity to Ca^2^in OL. From this perspective, one cannot rule out a possible metabolic effect of AT_1_blockade, providing greater energy efficiency from improved combustion of macronutrients, especially lipids.^24 , 36^ Excessive fatty acids supply may promote greater mitochondrial activity, stimulating mechanisms regarding increased Ca^2^handling.^19 , 23^ In a previous experiment, intervention with losartan resulted in the inhibition of molecular mechanisms of myocardial insulin resistance, improving contractile heart performance in obese rats by cafeteria diet.^11^ Recently, AT_1_blockade resulted in improved mitochondrial function in obese insulin-resistant rats.^37^

From this perspective, the clinical repercussions of the findings of this study are diverse. RAAS activation conditions have been associated with metabolic disorders and heart disease.^23^ In this study, important contractile disorders were shown, which could be the focus of interventions for cardiovascular treatment in obese patients.

However, isolated effects of dietary variables as a cause of cardiac remodeling cannot be ruled out, although these effects have been improved with AT_1_antagonism. In a previous study,^8^ increased lipid consumption was shown to be directly related to characteristics of cardiovascular response in obesity. Therefore, this is an important study limitation and new investigations should be developed to better clarify the isolated role of saturated and unsaturated fatty acids in this experimental model.

## Conclusion

In conclusion, high-fat diet-induced obesity promotes cardiac remodeling, sustained by ventricular hypertrophy and myocardial dysfunction. Considering that Losartan attenuated most of these disorders, the initial hypotheses of this investigation was confirmed, according to which the AT_1_receptor stimulation is associated with impaired myocardial function in obese rats.
